# Pitch Proposal: Recommenders with a Mission - Assessing Diversity in News Recommendations

**DOI:** 10.1007/978-3-030-65965-3_38

**Published:** 2020-12-09

**Authors:** Sanne Vrijenhoek, Natali Helberger

**Affiliations:** 5 University of Sydney, Sydney, NSW Australia; 6 Monash University, Clayton, VIC Australia; 7 University of Bari Aldo Moro, Bari, Italy; 8 University of Bari Aldo Moro, Bari, Italy; 9 University of Guelph, Guelph, ON Canada; 10 University of Caen Normandy, Caen, France; 11 University of Pisa, Pisa, Italy; 12 Norwegian University of Science and Technology, Trondheim, Norway; 13 University of Porto, Porto, Portugal; 14 UPC BarcelonaTech, Barcelona, Spain; 15 University of Porto, Porto, Portugal; 16 Fraunhofer IAIS, St. Augustin, Germany; 17 Royal Holloway University of London, Egham, UK; 18 University of Lisbon, Lisbon, Portugal; 19 University of Bari Aldo Moro, Bari, Italy; 20 University of Lisbon, Lisbon, Portugal; 21 University of Bari Aldo Moro, Bari, Italy; 22 ICAR-CNR, Rende, Italy; 23 University of Naples Federico II, Naples, Italy; 24 University of North Carolina, Charlotte, NC USA; 25 Australian National University, Canberra, ACT Australia; 26 Leibniz University Hannover, Hannover, Germany; 27 Technical University of Dortmund, Dortmund, Germany; 28 University of Southern Denmark, Odense, Denmark; 29 University of Pisa, Pisa, Italy; 30 Warsaw University of Technology, Warsaw, Poland; 31 ISTI-CNR, PISA, Italy; 32 Berlin Institute of Technology, Berlin, Germany; 33 Berlin Institute of Technology, Berlin, Germany; 34 Norwegian University of Science and Technology, Trondheim, Norway; University of Amsterdam, Amsterdam, The Netherlands

## Abstract

By helping the user find relevant and important online content, news recommenders have the potential to fulfill a crucial role in a democratic society. Simultaneously, recent concerns about filter bubbles, fake news and selective exposure are symptomatic of the disruptive potential of these digital news recommenders. Recommender systems can make or break filter bubbles, and as such can be instrumental in creating either a more closed or a more open internet. This document details a pitch for an ongoing project that aims to bridge the gap between normative notions of diversity, rooted in democratic theory, and quantitative metrics necessary for evaluating the recommender system. Our aim is to get feedback on a set of proposed metrics grounded in social science interpretations of diversity.

## Introduction

News recommender algorithms have the potential to fulfill a crucial role in democratic society. By filtering and sorting information and news, recommenders can help users to overcome maybe the greatest challenge of the online information environment: finding and selecting relevant online content. Informed by data on what people like to read, what their friends like to read, what content sells best, etc., recommenders use machine learning and AI techniques to make ever smarter suggestions to users [4, 12, 13, 20]. With this comes the power to channel attention and shape individual reading agendas and thus new risks and responsibilities. Recommender systems can be pivotal in deciding what kind of news the public does and does not see. Depending on their design, recommenders can either unlock the diversity of online information [7, 15] for their users, or lock them into boring routines of “more of the same”, or in the worst case into so-called filter bubbles [16] and information sphericules. But what exactly is diverse? As central as diversity is to many debates about the optimal design of news recommenders, as unclear it is what diverse recommender design actually entails. In the growing literature about diverse recommender design, a growing gap between the computer science and the normative literature can be observed. For news recommenders to be truly able to unlock the abundance of information online and inform citizens better, it is imperative to find ways to overcome the fundamental differences in approaching and conceptualizing diversity. There is a need to reconceptualise this central but also elusive concept in a way that both does justice to the goals and values that diversity must promote, as well as facilitates the translation of diversity into metrics that are concrete enough to inform algorithmic design. This pitch details the normative theory underlying our approach to evaluating diverse recommender systems, and five proposed metrics that follow from this theory. Our goal is to obtain feedback during the workshop on the applicability and explainability of these metrics, before we proceed with their operationalization in follow-up research.

## Theory

Before we define more quantitative metrics to assess diversity in news recommendation, we first offer a conceptualization of diversity. Following the definition of the Council of Europe, diversity is not a goal in itself, it is a concept with a mission, and it has a pivotal role in promoting the values that define us as a democratic society. These values may differ according to different democratic approaches. This article builds on a conceptualisation of diversity in recommendations that have been developed by [7]. [7] combines the normative understanding of diversity, meaning what should diverse recommendations look like, with more empirical conceptions, meaning what is the impact of diverse exposure on users. There are many theories of democracy, but we concentrated on 4 of the most commonly used theories when talking about the democratic role of the media: *Liberal*, *Participatory*, *Deliberative* and *Critical* theories of democracy (see also [2, 3, 10, 19]). It is important to note that no model is inherently better or worse than another. Which model is followed is something that should be decided by the media companies themselves, following their mission and dependent on the role they want to play in a democratic society.

### The Liberal Model

In liberal democratic theory, individual freedom, including fundamental rights such as the right to privacy and freedom of expression, dispersion of power but also personal development and autonomy of citizens stands central. Under such liberal perspective, diversity would entail a user-driven approach to diversity that reflects citizens interests and preferences not only in terms of content, but also in terms of for example style, language and complexity. A liberal recommender is required to inform citizens about prominent issues, especially during key democratic moments such as election time, but else it is expected to take little distance from personal preferences. It is perfectly acceptable for citizens to be consuming primarily cat videos and celebrity news, as long as doing so is an expression of their autonomy.

### The Participatory Model

An important difference between the liberal and the participatory model of democracy is what it means to be a good citizen. Under participatory conceptions, the role of (personal) freedom and autonomy is to further the common good, rather than personal self-development [8]. Accordingly, the media, and by extension news recommenders must do more than to give citizens ‘what they want’, and instead provide citizens with the information they need to play their role as active and engaged citizens [1, 6, 9, 11], and further the participatory values, such as inclusiveness, equality, participation, tolerance. Here the challenge is to make a selection that gives a fair representation of different ideas and opinions in society, while also helping a user to gaining a deeper understanding, and feeling engaged, rather than confused. This means that diversity is not only a matter of the diversity of content, but also of communicative styles. What would then characterize diversity in a participatory recommender are, on the one hand, active editorial curation in the form of drawing attention to items that citizens ‘should know’, taking into account inclusive and proportional representation of main political/ideological viewpoints in society and a heterogeneity of styles and tones, possibly also emotional, empathetic, galvanizing, reconciliatory.

### The Deliberative Model

The participatory and the deliberative models of democracy have much in common (compare [5]). Also in the deliberative or discursive conceptions of democracy, community and active participation of virtuous citizens stands central. One of the major differences is that the deliberative model operates on the premise that ideas and preferences are not a given, but that instead we must focus more on the process of identifying and negotiating and, ultimately, agreeing on different values and issues [5, 10]. Diversity in the deliberative conception has the important task of confronting the audience with different and challenging viewpoints that they did not consider before, or not in this way [14]. Concretely, this means that a deliberative recommender (or recommendation) should include a higher share of articles presenting various perspectives, diversity of emotions, range of different sources; it should strive for equal representation, including content dedicated to different ethnic, linguistic, national groups, as well as on recommending items of balanced content, commentary, discussion formats, background information, as well as a preference for rational tone, consensus seeking, inviting commentary and reflection.

### The Critical Model

A main thrust of criticism of the deliberative model is that it is too much focused on rational choice, on drawing an artificial line between public and private, on overvaluing agreement and disregarding the importance of conflict and disagreement as a form of democratic exercise [11]. The focus on reason and tolerance muffles away the stark, sometimes shrill contrasts and hidden inequalities that are present in society, or even discourage them from developing their identity in the first place. Good and diverse critical recommenders hence do not simply give people what they want. Instead, they actively nudge readers to experience otherness, and draw attention to the marginalised, invisible or less powerful ideas and opinions in societies. And again, it is not only the question of what kinds of content are presented but also the how: whereas in the deliberative and also the participatory model, much focus is on a rational, reconciling and measured tone, critical recommenders would also offer room for alternative forms of presentations: narratives that appeal to the ‘normal’ citizen because they tell an everyday life story, emotional and provocative content, even figurative and shrill tones - all with the objective to escape the standard of civility and the language of the stereotypical “middle-aged, educated, blank white man” [21].

## Metrics

**Table 1.** Overview of the different models and expected value ranges for each metric. The column refer to Calibration (topic) (Cal t), Calibration (style) (Cal s), Fragmentation (Frag), Affect, Representation (Rep), and Inclusion (Inc).

	Cal t	Cal s	Frag	Affect	Rep	Inc
Liberal	High	High	High	–	–	–
Participatory	Low	High	Low	Medium	Reflective	Medium
Deliberative	–	–	Low	Low	Equal	–
Critical	–	–	–	High	Inverse	High

The democratic models described in the previous section lead to different expectations for recommender systems in terms of diversity. In this section, we propose five novel metrics for assessing diversity in news recommendations, that follow directly from these expectations: *Calibration*, *Fragmentation*, *Affect*, *Representation* and *Inclusion*. Table 1 provides an overview of the different models and their expected value ranges for each of the different metrics.

### Calibration

The *Calibration* metric expresses to what extent the issued recommendations reflect the user’s preferences, and is a well-known metric in traditional recommender system literature [18]. However, we extend our notion of Calibration to not only include topicality. News recommendations can also be tailored to the user in terms of article style and complexity, allowing the reader to receive content that is attuned to their information needs and processing preferences. This may be split up within different topics; a user may be an expert in the field of politics but less so in the field of medicine, and may want to receive more complex articles in case of the first, and less in case of the second.

**In the Context of Democratic Recommenders.** For the Liberal model we expect high Calibration, both in terms of style and topicality. For the Participatory model we expect low topic Calibration, but high style Calibration.

### Fragmentation

News recommender systems create a recommendation by filtering from a large pool of available news items. By doing so they may stimulate a common public sphere, or create smaller and more specialized ‘bubbles’. This may occur both in terms of topics recommended, which is the focus of the Fragmentation metric, and in terms of political orientation, which will be later explained in the Representation metric. Fragmentation specifically compares differences in recommended news stories *among* users; the smaller the difference, the more we can speak of a joint agenda. It is important here to focus on news story chains rather than individual articles, to account for sets of articles that may be written in a different style or from a different perspective, but that ultimately discuss the same issue.

**In the Context of Democratic Recommenders.** For both the Participatory and Deliberative models we expect low Fragmentation. For the Liberal model we expect a higher score.

### Affect

The way in which an article is written may affect the reader in some way. An impartial article may foster understanding for different perspectives, whereas an emotional article may activate them to undertake action. The *Affect* metric aims to capture this by measuring the strength of emotions expressed in an article. In the context of democratic news recommenders a dimensional [17] approach is taken; what matters is the degree of ‘activation’ that is conveyed, not whether this happens in the positive or negative spectrum. It must be noted that it is less interesting what the feelings of the article’s author are, and more how these feelings affect the reader. However since this is very difficult to measure or predict we hold to the assumption that a strongly emotional article will also cause strong emotions in a reader.

**In the Context of Democratic Recommenders.** In the Deliberative model we aim for neutrality, and therefore low Affect. In the Participatory model a slightly wider value range is expected; some affective content is acceptable, but nothing too extreme. The Critical model however focuses specifically on affective content, and high values should be expected.

### Representation

One of the most intuitive interpretations of diversity focuses on its level of Representation, or the question whether the issued recommendations provide a good balance of different opinions and perspectives. Here we care more about what is being said than who says it, which is the goal of the last metric Inclusion.

**In the Context of Democratic Recommenders.** To define what it means to provide “a good balance” of opinions, one needs to refer back to the different models and their goals. The Participatory model aims to provide a good reflection of “the real world”. The news recommendations therefore need to have a larger share in the Representation for the more prevalent opinions in society. On the other hand, the Deliberative model aims to provide a complete overview of all opinions without one being more prevalent than the other.

### Inclusion

Where Representation is largely focused on the explicit content of a perspective (the *what*), Inclusion is more concerned with the person holding it (the *who*), and specifically whether this person or organisation is one of a minority group or an otherwise marginalised group that is more likely to be underrepresented in the mainstream media. What exactly entails a minority is about as vaguely defined as the concept of diversity itself: it may for example be related to ethnicity, gender, language group, religion, sexuality, disability.

**In the Context of Democratic Recommenders.** In the Critical model we aim for a high Inclusion score. The Participatory model fosters tolerance and empathy, and therefore we expect a slightly larger than average Inclusion.

## Conclusion

At the basis of our work is that we believe diversity is not a single absolute, but rather an aggregate value with many aspects. In fact, we argue that what constitutes ‘good’ diversity in a recommender system is largely dependent on its goal, which type of content it aims to promote, and which model of the normative framework of democracy it aims to follow. As none of these models is inherently better or worse than the others, we believe that a media company should take a normative stance and evaluate their recommender systems accordingly.
